# Intermolecular forces at the interface between NPs and biological systems

**DOI:** 10.1016/j.bbrep.2025.102300

**Published:** 2025-10-11

**Authors:** Eder Linares Vargas

**Affiliations:** University of Atlántico, Faculty of Education Bachelor's Degree Program in Natural Sciences, Colombia

**Keywords:** Nanoparticles, Nano-bio system, Physicochemical properties

## Abstract

Nanoparticles used for theranostic purposes interact with the cell membrane, thereby establishing a series of nanoparticle/biological system interfaces. These interactions often lead to biocompatible or bioadverse outcomes, as previously referenced. The development of predictive relationships between the nanoparticle and the biological system is determined by the physicochemical properties of the nanomaterial, such as shape, surface characteristics, roughness, size, and surface coating, among others. The objective of this article is to determine how the physicochemical properties of nanoparticles influence their interactions with biological systems, particularly at the nano-bio interface, aiming to enhance their effectiveness in biomedical applications such as drug delivery and cancer therapies. It focuses on the detailed analysis and exposition of the interactions between nanoparticles (NPs) and biological systems, especially at the nano-bio interface. No original experiments are presented; rather, it provides a compilation, analysis, and discussion of previous studies with an explanatory approach. It is concluded that studying the relationships at the interface (membrane/cell assembly) allows us to understand the influences these have on the final fate of these nanostructures, making them more efficient and effective in the fight against cancer. It is concluded that studying the relationships at the interface (membrane/cell assembly) allows us to understand the influences these have on the final fate of these nanostructures, making them more efficient and effective in the fight against cancer. Recent advances have provided strong evidence supporting these perspectives [17]; [18]; [24]; [25], showing how exosomal corona formation, calcium-functionalized nanomaterials, and smartly designed nanostructures are reshaping the understanding of cancer nanotherapy.

## Highlights

• Nanoparticles interact with cells at the nano–bio interface.
• Van der Waals, electrostatic, and solvation forces drive NP adhesion.
• NP size, surface, and charge influence biological interactions.
• Protein corona modifies NP behavior in biological environments.
• Insights support the design of predictive drug delivery systems.

## 1 Introduction

A biological system can be considered a complex network of entities that are physiologically essential to life. Biological systems exist on scales ranging from mesoscopic to nanoscopic, with cells being a clear example. The cell is regarded as the morphological and functional unit of all living organisms [1]. Like all living organisms, cells respond to stimuli. Most cells are covered with specific receptors that enable them to interact with substances in their surrounding environment. It is noteworthy that they do not respond the same way to various stimuli, as they possess receptors for hormones and extracellular materials. These receptors, located on the cell membrane, essentially serve as gateways through which different external agents trigger highly specific responses. The cell membrane is a lipid bilayer primarily composed of phospholipids that separates the intracellular environment from the extracellular medium [2].

The cell membrane is composed of a variety of proteins, which are generally classified into five main functional groups: receptor, recognition, enzymatic, anchoring, and transport proteins. Receptor proteins have the ability to recognize and bind specific molecules. This type of protein can identify a hormone, neurotransmitter, or even a nutrient necessary for cellular function. The molecule that binds to the receptor is called a ligand [3]. On the other hand, recognition proteins are typically glycoproteins responsible for tagging and identifying cells. Enzymatic proteins catalyze various chemical reactions, either synthesizing or breaking down biological molecules. Finally, transport proteins regulate the movement of hydrophilic molecules across the plasma membrane. Some of these are known as channel proteins because they form channels or pores through which water molecules or ions can pass across the membrane [[1], [2], [3], [4], [5], [6], [7], [8], [9], [10], [11], [12], [13], [14], [15], [16], [17], [18], [19], [20], [21], [22], [23], [24], [25], [26], [27], [28], [29], [30], [31], [32], [33], [34], [35], [36], [37], [38], [39], [40], [41], [42], [43], [44]].

### 1.1 Nanoparticles

A particle is classified as a nanoparticle when its dimensions are less than 100 nm. Nanoparticles (NPs) have recently emerged as a highly fruitful area of research due to their numerous applications in fields such as nanobiomedicine [5,6]. NPs can be synthesized using various techniques depending on the source material, and they may exhibit diverse shapes and sizes. Nevertheless, they retain the distinctive property of being functionalizable, enabling the attachment of a wide range of ligands, including folic acid, specific antibodies, polymers such as chitosan, therapeutic agents like doxorubicin, and genetic material. Due to these unique features, NPs have attracted considerable interest in the field of nanomedicine [7].

The use of nanoparticles offers several advantages for drug delivery, mainly due to their size and biodegradability, which are critical to their functionality. Their small dimensions allow them to penetrate biological tissues efficiently [8]. Moreover, their size favors intravenous administration by preventing aggregation phenomena [9,10]. Nanotechnology, in general, has evolved into a highly promising research domain. Within it, a specialized branch focuses on the application of nanotechnology to biological systems. This area explores the interactions between nanomaterials and biological entities, where identifying the physicochemical properties that predict such interactions becomes essential—this being the central objective of the present study.

### 1.2 Physicochemical properties of NPs as a function of size

It is well established that predictive relationships between nanoparticles and biological systems are governed by the physicochemical properties of the nanomaterials, including shape, surface characteristics, roughness, size, and surface coating, among others. Below are some key properties influenced by particle size. The size of NPs plays a pivotal role in determining a series of intrinsic physicochemical properties derived from the nature and dimensions of the material. Their high surface energy imparts a degree of instability that significantly affects their behavior [11]. Undoubtedly, size is a critical factor influencing the physicochemical characteristics of nanoparticles, which must be understood and predicted, as these properties ultimately affect biodistribution, absorption, and membrane receptor binding [12]. NP size is also affected by a phenomenon known as aggregation, which further contributes to instability. This natural tendency influences both the final shape and size of the particles, thereby altering their functional properties. Hence, preventing aggregation is crucial [13]. Another key factor is the Z-potential, which directly impacts the stability and physicochemical behavior of the nanomaterial. Fluctuations in this potential can destabilize the material, significantly promoting aggregation [14].

## 2 Methodological approach and scope of the analysis

The methodology employed in this study is theoretical, analytical, and bibliographic in nature. It is based on an exhaustive review of up-to-date scientific literature aimed at examining the interactions between nanoparticles (NPs) and biological systems, with a particular focus on the nano-bio interface. This work compiles and discusses previous research on the physicochemical properties of nanomaterials, such as size, shape, surface roughness, Z-potential, and aggregation mechanisms.

The theoretical framework incorporates advanced physicochemical models to describe the fundamental forces that govern these interactions—namely, van der Waals, electrostatic, and solvation forces—explained through mathematical expressions derived from quantum mechanics, thermodynamics, and molecular physics. Furthermore, the behavior of NPs in complex biological environments is analyzed, considering effects such as protein corona formation, charge shielding described by the Debye length, and solvation dynamics in biological fluids such as blood. This article does not involve original experimental work. Instead, it adopts an explanatory and predictive approach to analyze how these interactions influence nanoparticle adhesion, stability, biodistribution, and internalization. The study is grounded in previous scientific work with solid theoretical and experimental backing, allowing the construction of a conceptual framework to guide future biomedical applications—particularly in the design of drug delivery systems and targeted therapies.

### 2.1 Dynamic interactions at the NP–biological system interface

The interaction between NPs and biological systems initially occurs at the so-called nano-bio interface—namely, the surface of the nanomaterial in contact with the biological entity. Within this interface, biophysical and biochemical processes take place that may exhibit predictive characteristics. This section aims to describe several dynamic interactions that occur when the nanoparticle assembly engages with a biological system.

In fact, the nano-bio interface can be characterized by three main phases of biophysical interaction: one dependent on the physicochemical composition of the nanoparticle surface, another resulting from the interaction of NPs with the surrounding medium, and finally, the actual contact region between the NP and the biological system [15].

These interactions have been reported to result in phenomena such as protein corona formation, particle wrapping, intracellular uptake, and biocatalytic processes, which may lead to either biocompatible or bioadverse outcomes. Furthermore, biomolecules can induce phase transformations, free energy release, surface restructuring, or even dissolution at the nanoparticle interface. This highlights the importance of understanding such interactions, as they can serve as predictive tools in the evaluation of nanoparticle behavior in biological environments [16].

In line with these considerations, Önal Acet et al. [17] provided the first evidence that exosomes can form an exosomal corona around Fmoc-lysine-based nanomaterials, significantly reducing nanotoxicity and altering their physicochemical behavior. Similarly, Dikici et al. [18] demonstrated that calcium-functionalized dipeptide nanomaterials not only maintain high biocompatibility and physiological stability but also achieve precise zeta potential control, confirming their potential as safe carriers for gene delivery and drug release.

### 2.2 Dynamics at the nano-bio interface

Typical interactions between nanoparticles (NPs) immersed in liquid media (such as water or blood) and biological systems primarily involve van der Waals (VDW) forces, electrostatic forces, and solvation forces. VDW forces originate from quantum mechanical interactions within the nano-bio system. Small fluctuations induce polarization in the nanoparticle, creating a dipole moment that affects nearby atoms, leading to attractive or repulsive forces that are highly dependent on the separation distance.

Mathematically, the potential energy associated with van der Waals forces can be expressed as:

$U\left(r\right)=-\frac{C}{r^{6}}$ where *C* is a constant that depends on the molecular properties of the interacting species, and *r* is the distance between them [19,20].

van der Waals (VDW) forces are relatively weak compared to some chemical bonds; however, they play a critical role across multiple disciplines including molecular chemistry, biology, physics, and nanotechnology. These forces involve interactions between surfaces, molecules, and atoms, and arise from polarization fluctuations governed by quantum mechanics. The potential between molecules includes a repulsive term that prevents collapse and an attractive term, which can be represented by the Lennard-Jones potential model:

$U\left(r\right)=4\varepsilon \left[{\left(\frac{\sigma }{r}\right)}^{12}-{\left(\frac{\sigma }{r}\right)}^{6}\right]$ where ε is the depth of the potential well, and σ is the distance at which the potential energy equals zero [21].

The nature of van der Waals forces is associated with three types of interactions: London dispersion forces, Keesom dipole–dipole interactions, and Debye induced dipole interactions. London dispersion forces are particularly relevant for nonpolar nanoparticles and arise from the temporal correlation of instantaneous dipoles. The interaction energy of this type of force is modeled as:

$U_{London}\alpha -\frac{\alpha_{1}\alpha_{2}}{r^{6}}$ where α_1_ and α_2_ are the polarizabilities of the interacting molecules. On the other hand, the permanent dipole–dipole interaction follows the expression:

$U_{Keesom}-\frac{\mu_{1}^{2}\mu_{2}^{2}}{\left(4\pi \varepsilon_{0}k_{B}T\right)r^{6}}$ where μ_1_ and μ_2_ are the dipole moments of the molecules, ε_0_ is the vacuum permittivity, $k_{B}$ is the Boltzmann constant, and *T* is the absolute temperature. Finally, the Debye induced dipole interaction is described as: $U_{Debye}=-\frac{\mu^{2}\alpha }{r^{6}}$ where μ is the dipole moment of one molecule and α is the polarizability of the other ([22], pp. 368–376).

In the second case, represented by electrostatic forces, these arise from surface charges that inevitably develop on nanoparticles. Such charges may originate from the dissociation or displacement of functional groups, resulting in repulsive forces between particles. The electrostatic potential energy between two point charges, *q*_*1*_ and *q*_*2*_, is expressed as:

$U_{elect}=\frac{1}{4\pi \varepsilon }\frac{q_{1}q_{2}}{r}$ where ε is the permittivity of the medium in which the nanoparticles are suspended.

Finally, in the case of solvation, it arises when a nanoparticle (NP) interacts with a solvent, thereby stabilizing solute species and preventing aggregation. A key parameter used to describe these effects is the solvation free energy, ΔG_sol_, which determines the stability of the NP in the liquid medium. Solvation also plays a crucial role in the interaction between hydrophilic inorganic nanoparticles and biological systems. Solvent molecules can form protective steric layers around the NP, which can be described using the hydrodynamic radius, Rᴴ, dependent on both the particle radius and the thickness of the solvation layer.

The general equation for the solvation free energy is expressed as:

$$\Delta G_{\text{sol}}=\Delta G_{\text{cav}}+\Delta G_{\mathrm{int}}+\Delta G_{\text{pol}}+\Delta G_{\text{disp}.}$$

ΔG_cav_ is the free energy required to create a cavity in the solvent to accommodate the solute.
ΔG_int_ represents specific interactions such as hydrogen bonding between the solute and the solvent.
ΔG_pol_ is the contribution due to solute polarization by the solvent (significant in polar solvents).
ΔG_disp._ is the contribution from van der Waals dispersion forces between the solute and the solvent.

Ionic forces in the nano-bio interaction can reduce protein solubility in aqueous media. In biological fluids, the ionic strength is approximately 150 mM, indicating that resting surface charges can be effectively screened, thereby significantly diminishing the effects of van der Waals forces. The extent of electrostatic screening is described by the Debye length, λ_ᴰ_, given by: $\lambda_{D}=\sqrt{\frac{\varepsilon k_{B}T}{2N_{A}e^{2}I}}$ where $N_{A}$ is Avogadro's number, *I* is the ionic strength of the medium, and *e* is the elementary charge [23].

## 3 Discussion

The interaction between nanoparticles (NPs) and biological systems is governed by the combination of van der Waals (VDW), electrostatic, and solvation forces. Each of these forces plays a critical role in determining the stability, aggregation, and adhesion of NPs to biological cells. VDW forces, as described by the Lennard-Jones potential, represent a balance between attractive and repulsive terms that define the equilibrium distance between NPs and biomolecules on the cell surface. However, the presence of surface charges on NPs introduces electrostatic effects that can significantly alter this interaction.

**Fig. 1:**
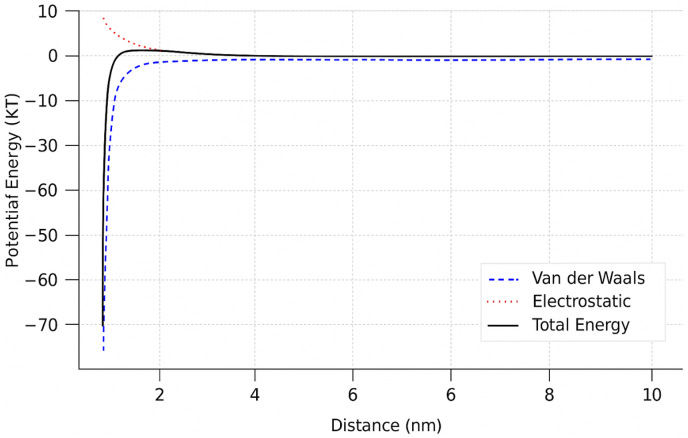
Interaction between van der Waals and electrostatic forces.

Mathematically, the competition between VDW and electrostatic forces can be analyzed by considering the superposition of their respective potentials. If a charged NP approaches a cell membrane with an opposite charge, the total interaction energy is given by the sum of the VDW and Coulomb terms:

$U_{total}=U_{VDW}+U_{elec}=-\frac{C}{r^{6}}+\frac{1}{4\pi \varepsilon }\frac{q_{1}q_{2}}{r}$ The NP may experience an energy barrier that prevents its direct adhesion to the cell, thereby promoting greater dispersion in the medium. On the other hand, if the electrostatic interaction is attractive (*q*_*1*_*q*_*2*_ < 0), the NP can approach more easily, favoring adsorption onto the cell membrane. The following graph illustrates the interaction between these two forces (see Fig. 1).

The graph illustrates the interaction between van der Waals and electrostatic forces: the blue (dashed) curve represents the van der Waals interaction, which is attractive and decays rapidly with distance $\alpha \frac{1}{r^{6}}$. The red (dotted) curve represents the electrostatic interaction, which may be either attractive or repulsive and follows a distance dependence of $\alpha \frac{1}{r}$. The black curve shows the total interaction energy, resulting from the sum of both forces. When the total energy exhibits a minimum, NP adhesion to the membrane is favored. If an energy barrier is present, the NP may remain dispersed in the medium rather than adhering.

On the other hand, in biological environments such as blood plasma, the presence of ions induces electrostatic screening, thereby reducing the range of electrostatic forces and promoting the dominance of VDW forces. The Debye length, given by:

$\lambda_{D}=\sqrt{\frac{\varepsilon k_{B}T}{2N_{A}e^{2}I}}$ determines the distance at which charges become effectively neutralized. In fluids with high ionic strength (large *I*), the screening effect is stronger, reducing the influence of electrostatic interactions on NP adhesion to cells.

**Fig. 2:**
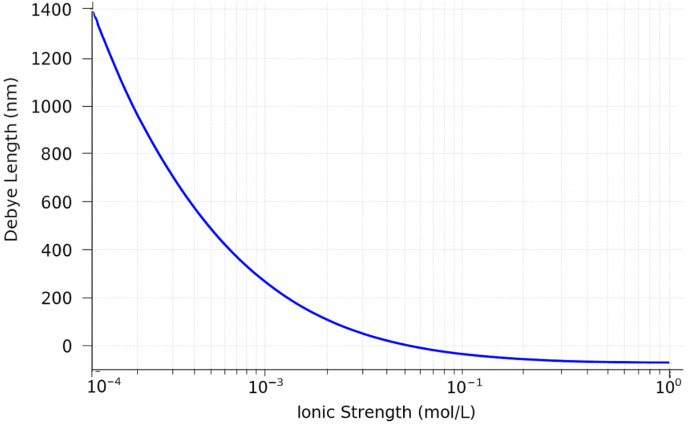
Effect of Debye screening.

The graph (see Fig. 2) shows that as the ionic strength *I* increases, the Debye length *λ*_*ᴰ*_ decreases, indicating stronger electrostatic screening. In media with high ionic strength (such as blood plasma), the screening is significant, and electrostatic interactions become less effective over long distances. In contrast, in low ionic strength environments, electrostatic forces can act over greater distances.

In the case of solvation, steric effects are introduced that may modify the interaction between NPs and biological systems. In aqueous media, the formation of a solvation layer around the NP—characterized by the hydrodynamic radius *R*_*ᴴ*_ - creates an energetic barrier that prevents uncontrolled aggregation. In this context, the solvation free energy *ΔG*_*sol*_ determines the nanoparticle's stability in the biological fluid and its propensity to interact with cellular structures.

The adhesion between nanoparticles (NPs) and biological systems depends on multiple factors, including surface proximity, as well as the geometry and chemical nature of the cell membrane. As NPs approach the cell surface, van der Waals (VDW) forces become dominant, facilitating adhesion. However, the shape and size of NPs also influence these interactions, as a higher surface-to-volume ratio increases the contact area and, consequently, the intensity of VDW forces. Surface roughness of either the NP or the cell may further enhance these interactions, promoting greater adsorption onto the biological membrane.

In addition to VDW forces, electrostatic interactions play a key role in NP adhesion and stability. The surface charge of nanoparticles—determined by their composition and synthesis method—can result in attractive or repulsive interactions with the cell membrane, which is generally electronegative due to the presence of phosphatidylserine and other phospholipids. Positively charged NPs are more readily internalized, a factor of critical relevance in the design of drug delivery systems. However, in biological media such as blood, the surface charge of NPs can be altered by the adsorption of proteins, thereby modifying their electrostatic behavior and biodistribution.

The colloidal stability of NPs in a biological medium is also regulated by electrostatic forces. A well-distributed surface charge helps prevent NP aggregation, ensuring proper dispersion and effectiveness in therapeutic applications. In this context, the Debye length describes the intensity of electrostatic screening in media with high ionic strength, reducing the range of electrostatic interactions. This has direct implications for NP transport through biological fluids and their efficient arrival at target cells without forming aggregates that could reduce their functionality.

Solvation forces also play a fundamental role in nano-bio dynamics, influencing the stability and interaction of NPs with the cellular environment. In aqueous media, solvation affects NP dispersion and, consequently, their ability to adhere to the cell membrane. If an NP has a highly hydrophobic surface, its solubility in an aqueous medium will be low, potentially reducing its bioavailability. The solvation layer surrounding the NP also modulates its interaction with cellular receptors, which is crucial in targeted therapies.

In biomedical applications, solvation influences processes such as endocytosis and drug release. Adequate interaction between the NP and the cellular environment can facilitate the internalization of NPs by tumor cells and the subsequent controlled release of therapeutic agents. In this context, functionalizing NPs with specific ligands—such as antibodies or peptides—enhances their affinity for target cells, thereby improving treatment efficacy. However, unfavorable interactions with the solvent can induce toxicity or adverse responses, making it essential to optimize the physicochemical properties of NPs to ensure their biocompatibility.

Despite the advances described, significant gaps remain in our understanding of the dynamics at the nano-bio interface. These include: (i) understanding how the protein corona modulates cellular internalization processes; (ii) the influence of tumor microenvironment heterogeneity on the biodistribution of nanoparticles; and (iii) the lack of comprehensive predictive models that integrate molecular simulations with in vivo experimental validation. These gaps open clear avenues for future research, particularly aimed at the development of intelligent nanostructured platforms for personalized cancer therapies.

These findings align with broader trends in the field, where Gül et al. [24] emphasized the role of intelligently designed nanostructures in overcoming the limitations of conventional cancer therapies, particularly in aggressive tumors such as head and neck cancers. Complementarily, Yıldırım et al. [25] highlighted the latest advances in nanoplatform design—including liposomes, dendrimers, DNA origami, and metallic nanoparticles—underscoring how smart, biodegradable, and biocompatible carriers are paving the way toward more precise and personalized cancer treatments.

## 4 Conclusions

The dynamics at the nano-bio interface result from the interplay and competition among van der Waals, electrostatic, and solvation forces. While the former promote adhesion, electrostatic forces may either attract or repel nanoparticles (NPs) depending on their surface charge, and solvation influences NP stability and dispersion in biological media. Although robust theoretical models exist to describe these interactions, the complexity of biological environments introduces variations that must be carefully considered. Factors such as membrane flexibility, medium composition, and surface charge modification of NPs play a crucial role in determining their behavior. Understanding these interactions is essential for the development of nanotechnological strategies in targeted therapies and drug delivery systems.

The nano-bio interface encompasses a series of surface interactions in which these forces play a highly significant role. Such interactions largely govern the physicochemical properties of the material. For instance, the formation of protein coronas is a direct consequence of this dynamic interplay. The study of various interfaces calls for the establishment of new research directions, where understanding the influences of size, shape, surface characteristics, and other parameters—combined with the intent to employ NPs as nano-carriers for effective drug delivery—will support the selection of optimal strategies for the synthesis and characterization of these nanostructures.

## Fources of research funding

This research did not receive any specific grant from funding agencies in the public, commercial, or not-for-profit sectors.

## Declaration of competing interest

The authors declare that they have no known competing financial interests or personal relationships that could have appeared to influence the work reported in this paper.

## Data Availability

Data will be made available on request.
